# Left recurrent nerve lymph node dissection in robotic esophagectomy for esophageal cancer without esophageal traction

**DOI:** 10.1186/s12957-023-03117-3

**Published:** 2023-07-26

**Authors:** Tomonori Nakanoko, Yasue Kimura, Keita Natsugoe, Kentaro Nonaka, Sho Nambara, Qingjiang Hu, Ryota Nakanishi, Mitsuhiko Ota, Eiji Oki, Tomoharu Yoshizumi

**Affiliations:** Dept. of Surgery and Science, Kyushu University, 3-1-1, Maidashi, Higashi-Ku, Fukuoka City, 812-8582 Japan

**Keywords:** Robot-assisted thoracoscopic esophagectomy, Left recurrent nerve lymph node dissection, Left recurrent nerve palsy, No retraction with the esophagus

## Abstract

**Background:**

Because the robotic arm is located on the dorsal side of the patient, when the esophagus is pulled dorsally for the left recurrent nerve lymph node (LRLN) dissection, the robotic arm interferes with the surgical field. This made it difficult to prepare for the left recurrent lymph node dissection. We developed LRLN dissection in robotic surgery with natural space creation by physiological organ movement and evaluated the short-term results.

**Methods:**

In this retrospective study, we analyzed 102 cases of robot-assisted thoracoscopic subtotal esophagectomy (RATE) among radical subtotal esophagectomies performed between December 2018 and December 2022 using medical records. LRLN dissection is preceded by a dissection of the esophagus from the trachea. Leaving the esophagus on the vertebral side and away from the trachea resulted in a physiological elevation of the esophagus, providing space between the trachea and esophagus.

**Results:**

The thoracic surgery time in RATE was 181 (115–394) min. The number of LRLNs dissected was 4 (1–14). Six patients (6%) had a postoperative recurrence in the mediastinal lymph nodes. Seven patients (7%) had grade ≥ 1 left recurrent nerve palsy.

**Conclusions:**

LRLN dissection with RATE using natural space creation was performed safely with a sufficient number of dissected lymph nodes and little left recurrent nerve palsy.

**Supplementary Information:**

The online version contains supplementary material available at 10.1186/s12957-023-03117-3.

## Introduction

According to the national clinical database in Japan, recurrent nerve palsy in esophageal cancer surgery is still reported in about 11.1% of cases [1]. The causes are considered to be excessive physical stress due to heating, compression, and flexion [2]. Recurrent nerve palsy can cause postoperative pneumonia and dysphagia. As postoperative complications are a poor prognostic factor in esophageal cancer surgery, recurrent nerve palsy is one of the problems to be avoided [3, 4]. Especially in left recurrent lymph node dissection, the space between the upper esophagus and trachea is narrow, which requires sufficient working space for safe and radical dissection [5, 6]. Traction of the upper esophagus to the dorsal side of the patient is an effective maneuver for ensuring a large working space [6, 7]. RATE was performed in the supine position, with the robotic arms positioned directly over the dorsal side of the patient. The operation of preparing esophageal traction on the dorsal side would interfere with the robotic arms. To avoid this interference, we have investigated a surgical field development maneuver that does not involve the operation of esophageal traction from the patient’s dorsal side. We have developed and formalized a natural surgical field creation maneuver for the left recurrent nerve lymph node (LRLN) in RATE.

### Patients and methods

One hundred two cases of esophageal squamous cell carcinoma undergoing robot-assisted radical resection with McKeown esophagectomy from December 2018 to December 2022 were reviewed. The surgical and postoperative outcomes were retrospectively reviewed using data extracted from clinical records. Complications were defined as Clavien-Dindo grade ≥ 1.

### Surgical procedure

#### Patient positioning and port placement

The patient is intubated with a single lumen tube and left single lung ventilation with a blocker. The patient is placed in the left lateral decubitus position with the right arm elevated to the cranial side.

An 8-mm port is placed between the 3rd intercostal space at the midaxillary line, the 5th and 7th intercostal spaces at the posterior axillary line, and the 9th intercostal space slightly ventral to the subscapular angle. The 7th intercostal space is used as the camera port, the 1st arm uses the 9th intercostal space, and the 3rd and 4th arms use the 5th and 3rd intercostal spaces, respectively. A 12-mm trocar is added to the 6th intercostal space on the middle axillary line as an assist port. The robot is rolled in from the right side of the operating table. A 0-degree camera is used for the middle and lower mediastinal lymph node dissection, and a 30-degree camera is used for the upper mediastinal lymph node dissection. The arm and camera positions were not changed for upper mediastinal lymph node dissection.

### Maneuver of lymph node dissection surrounding the left recurrent nerve (Supplementary video)

In LRLN dissection, sufficient dissection space is considered necessary for adequate dissection and the avoidance of recurrent nerve palsy. In thoracoscopic subtotal esophagectomy, the azygos vein arch is resected and pulled dorsally, followed by prior dissection of the esophagus from the dorsal side, dissection from the tracheobronchial membrane, and then retraction of the esophagus from the dorsal side to provide space for LRLN dissection. However, in RATE, the process of retracting the esophagus should be performed between the robotic arms, which causes inconvenience due to the narrow space.

**Fig. 1:**
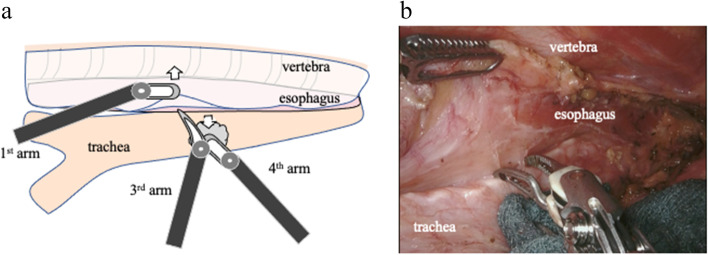
**a** Schematic illustration of “Step 1” of the left recurrent nerve lymph node (LRLN) dissection. The trachea is retracted clockwise by the 4th robotic arm. The pleura around the upper esophagus is retracted posteriorly by the 1st robotic arm, and the esophagus is gently removed from the tracheal membrane by the 3rd robotic arm. **b** Actual operative scene of “Step 1” of the LRLN dissection

Step 1 (Fig. 1): The azygos vein is preserved without resection. The pleura is cut along the run of the right vagus nerve toward the right subclavian artery. The esophagus is then dissected from the tracheal membrane. At this time, it should be separated sufficiently to the cranial side. The right lateral edge of the tracheal cartilage and connective tissue including the right recurrent nerve lymph node (RRLN) are mobilized, and then, the RRLN is dissected. The esophagus and the tracheobronchial space should be dissected to the cranial side to create mobility of the esophagus and pull it toward the vertebral body, facilitating the mobilization of connective tissue including the RRLN. While gently rotating the trachea clockwise with the 4th arm and pushing up and draining the esophagus dorsally with the 1st arm, the esophagus is detached from the trachea using the bipolar Maryland forceps on the 3rd arm. The esophagus should be completely transferred from the trachea to the cranial side (Supplementary Figure a).

**Fig. 2:**
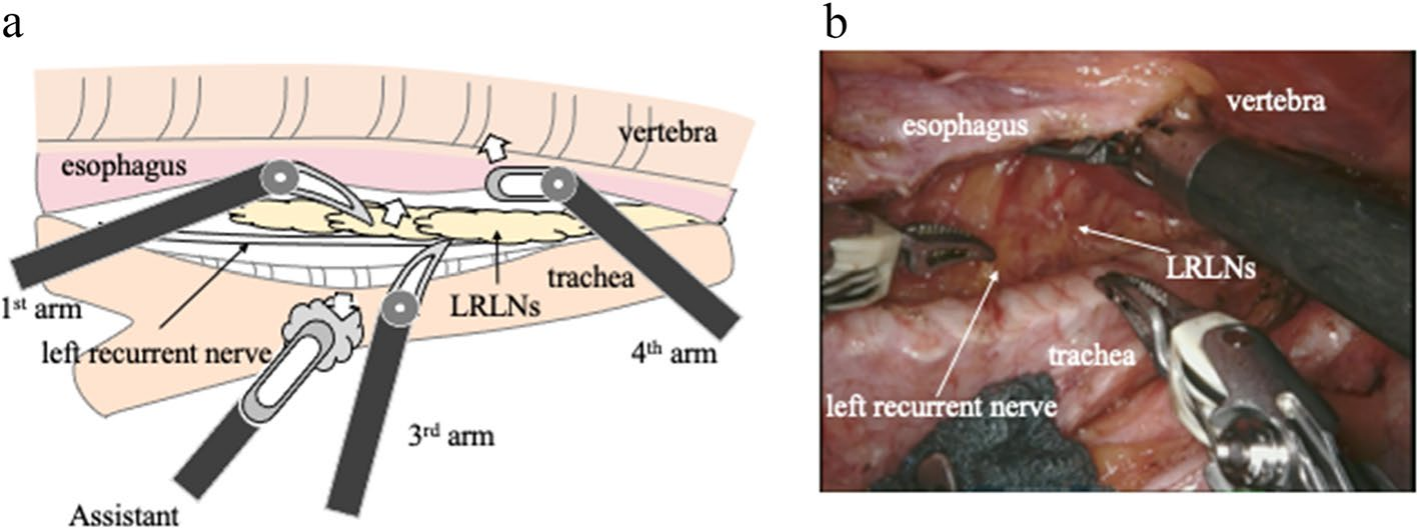
**a** Schematic illustration of “Step 2” of the left recurrent nerve lymph node (LRLN) dissection. The trachea is rotated clockwise by the assistant from the 6th intercostal port. Working space for lymph node dissection could be created naturally, and the esophagus could be partially retracted posteriorly with the 4th intercostal port. LRLNs were retracted by the 1st robotic arm and dissected from the surrounding tissue by the 3rd robotic arm. **b** Actual operative scene of “Step 2” of LRLN dissection

Step 2 (Fig. 2): After the first step, the trachea is rotated by an assistant. The esophagus remains attached to the vertebral side and to the vascular sheath surrounding the left subclavian artery, and the space between the esophagus and the trachea is naturally expanded. A working space for the dissection of the LRLN can be created physiologically and naturally. By pushing the esophagus dorsally with the 4th arm, more working space can be created. When the LRLN is mobilized from the left recurrent nerve sheath, lymph node dissection can be performed while checking the course of the left recurrent nerve fiber. The nerve branch to the esophagus should be cut with scissors, and the lymph nodes around the left recurrent nerve should be turned to the esophageal side. Flip up the entire LRLN from the left recurrent nerve. In this process, the esophageal branch of the left recurrent nerve is also transected; once the LRLN is moved to the esophageal side, the left recurrent nerve is transferred from the dorsal connective tissue, freeing it from the surrounding tissue (Supplementary Figure b).

**Fig. 3:**
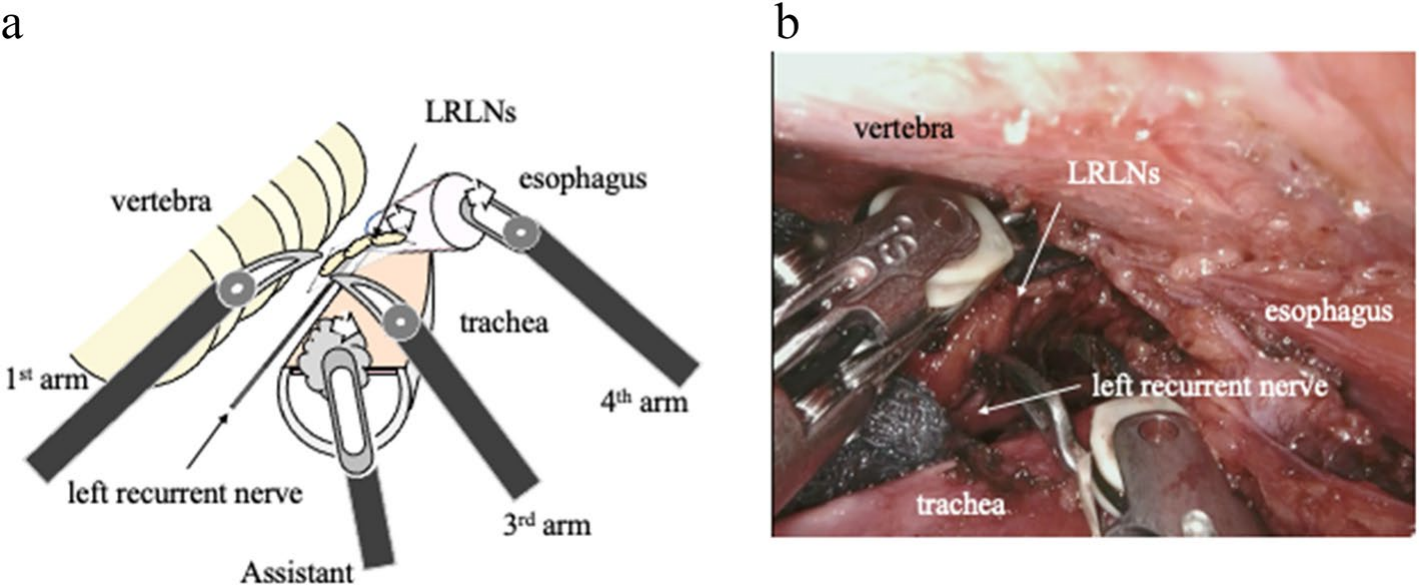
**a** Schematic illustration of “Step 3” of the left recurrent nerve lymph node (LRLN) dissection. After partial resection of the esophagus, the cranial side of the esophagus was retracted to the lateral side by the 4th arm of the robot to create a larger working space on the cranial side. The lymph nodes were gently retracted by the 1st robotic arm and removed from the surrounding tissue by the 3rd robotic arm. **b** Actual operative of “Step 3” of LRLN dissection

Step 3 (Fig. 3): The esophagus is separated from the vascular sheath surrounding the vertebral body and the left subclavian artery. Resection of the dorsal connective tissue is performed well up to the level where the left recurrent nerve is located. The thoracic duct is preserved on the side of the vascular sheath (Supplementary Figure c). A membrane will be formed between the esophagus and the vascular sheath by the connective tissue, including the LRLN. The esophagus is cut at the level of the aortic arch using a linear stapler. When the cranial side of the esophagus is pulled outward with the 4th arm, the LRLN is pulled outward along with the membrane created between the esophagus and the vascular sheath. At this time, the left recurrent nerve has already completed its separation from the surrounding connective tissue, and traction on the esophagus no longer exerts tension on the left recurrent nerve (Supplementary Figure d). By releasing the left recurrent nerve from the vascular sheath beforehand, the left recurrent nerve can be kept in its natural position without being pulled along with the retracted esophagus. The LRLN is located in the mesentery, which is composed of the esophagus and the vascular sheath, and the dissected lymph node is separated from the vascular sheath with the mesentery. Sufficient distance was created between the left recurrent nerve and the dissected lymph node.

On the other hand, on the cranial side, the left recurrent nerve penetrates the LRLN. In this area, after giving the LRLN from the tracheal side ahead of time, the connective tissue is detached from the dorsal esophagus to the level where the left recurrent nerve runs, and the LRLN is separated from the surrounding tissue with the nerve penetrating it. The LRLN is divided along the nerve run, and the LRLN is detached from the nerve and dissected. Finally, the LRLN attached to the esophageal side is removed from the esophagus and retrieved, and LRLN dissection can be completed.

## Results

The clinical background is shown in Table 1. The most common tumor site was the Mt in 55 (54%) male patients, and preoperative treatment was performed in 65 (64%) patients.

**Table 1:** Clinical background

	RATE (*n* = 102)
Age (range)	68 (44 – 83)
Sex (%)	
Male	77 (75)
Female	25 (25)
Tumor location (%)	
Upper	22 (22)
Middle	55 (54)
Lower	25 (24)
cT (%)	
1	34 (33)
2	18 (18)
3	46 (45)
4a	4 (4)
cN (%)	
0	51 (50)
1	27 (26)
2	23 (23)
3	1 (1)
cStage (%)	
I	37 (36)
II	16 (16)
III	47 (46)
IVA	2 (2)
Preoperative treatment	
None (%)	37 (36)
CT or CRT (%)	65 (64)

*RATE* Robot-assisted thoracoscopic esophagectomy, *CT* Chemotherapy, *CRT* Chemoradiotherapy

The outcomes of surgery are shown in Table 2. The time required for thoracic surgery was 181 (115–394) min. The number of LRLNs dissected was 4 (1–14), and the number of cases of recurrence in the mediastinal lymph nodes was 6 (6%).

**Table 2:** Surgical and postoperative outcomes

	RATE (*n* = 102)
Operation time (min)	
Thoracic surgery (range)	181 (115 – 394)
Blood loss (g) (range)	93 (1 – 530)
Number of Lymph node retrieved	
Upper mediastinal LNs (range)	10 (1 – 29)
LRLNs (range)	4 (1 – 14)
Recurrence in Mediastinal LNs (%)	6 (6)

*RATE* Robot-assisted thoracoscopic esophagectomy, *LN* Lymph Node, *LRLN* Left Recurrent Nerve Lymph node

Postoperative complications are shown in Table 3 and included pneumonia (*n* = 5; 5%), anastomotic leakage (*n* = 2; 2%), and recurrent nerve palsy (*n* = 7; 7%).

**Table 3:** Postoperative complications

	RATE (*n* = 102)
All _(%)_	22 (22)
Pneumonia (%)	5 (5)
Anastomotic leakage (%)	2 (2)
Left recurrent nerve palsy (%)	7 (7)

*RATE* Robot-assisted thoracoscopic esophagectomy

## Discussion

The left recurrent nerve is located between the left subclavian artery and the trachea. The left subclavian artery is encased in a vascular sheath, and the trachea and esophagus are encased in a visceral sheath. The left recurrent nerve and LRLN are encased in connective tissue encased in the visceral sheath. In the narrow space anterior to the left recurrent nerve is a soft tissue membrane containing the LRLN and the tracheoesophageal artery that supplies the esophagus and outer tracheal wall [8, 9].

Before dissecting the LRLN, the soft tissue, including the tracheoesophageal artery, is separated from the trachea and left recurrent nerve. This requires sufficient working space between the tracheal wall and the left recurrent nerve. One method has been reported to traction the esophagus to the patient’s dorsal side [6, 7].

In our approach, the esophagus is dissected from the tracheobronchial side, and the natural traction of the esophagus caused by keeping it connected to the vertebral side provides space for dissection of the LRLNs. Suspending the esophagus from the patient's dorsal side interferes with the robotic arm. The omission of suspending the esophagus would eliminate the need for working between robot arms. We also believe that excessive suspension of the esophagus leads to excessive tension on the left recurrent nerve. By omitting esophageal suspending, interference between the robotic arm and suspending field cannot happen, and excessive tension on the left recurrent nerve can be avoided. Since the esophagus has been sufficiently detached from the trachea to the cranial side in the first step, sufficient working space can be obtained in the second step by rolling the trachea by the assistant. If the working space is still narrow on the cranial side, the 4th arm can be used to push up the esophagus to further increase the working space. At this time, the adipose tissue, including the LRLN, can also be dissected from the trachea sufficiently to the cranial side. In the cranial dissection of the LRLN, the rotation of the trachea by the assistant plus the push-up of the esophagus with the 4th arm creates sufficient working space. Rotation of the trachea and further elevation of the esophagus by the 4th arm can provide an adequate field of view for dissection without elevation of the esophagus by external traction.

With the assistant rotating the trachea and the robotic arm helping to push the esophagus dorsally, the working space is further expanded, allowing radical and safe LRLN dissection. In the esophageal traction method, the esophagus is moved up the spine, which increases the working space, but excessive traction of the esophagus leads to dorsal traction of the mesentery, together with the left recurrent nerve, which may cause flexion of the nerve fibers and recurrent nerve palsy [2]. Excessive external traction with taping may lead to excessive tension and flexion to the left recurrent nerve. We believe that the esophagus can be pulled toward the vertebral body by preceding the separation of the esophagus from the trachea, thus providing sufficient working space without excessive traction.

After performing esophageal dissection from the trachea, a flip-up of the lymph node from the anterior aspect of the LRLN to the esophageal side is performed. If the dorsal esophagus is then dissected from the vascular sheath, the lymph node will be located within the mesentery composed of the esophagus and vascular sheath, and traction on the mesentery, including the lymph node, will not exert tension on the left recurrent nerve. The lymph node dissection can be completed while the mesentery containing the lymph node is retracted with the esophagus.

The less frequency of left recurrent nerve palsy in this study was caused by these three factors: without excessive esophagus traction, the dissection of the esophageal branch of the left recurrent nerve, and the dissection in the parallel direction to the nerve fiber. Preceding detachment from the trachea allows the esophagus to be naturally retracted toward the vertebral body, and the addition of a tracheal rotation provides sufficient working space. We believe that this natural working space, combined with the characteristics of the robot, such as a high degree of flexibility in the range of motion of the joints, will enable safer lymph node dissection. Leaving the esophagus on the vertebral side also limits the range of motion of the esophagus to the vertebral body. By resecting the esophageal branch of the left recurrent nerve prior to dissection and detaching the left recurrent nerve from the dorsal connective tissue, the left recurrent nerve will not be pulled together when the esophagus is pulled laterally, thereby avoiding force tension on the left recurrent nerve.

The number of lymph nodes retrieved in this study, the incidence of recurrent nerve palsy, and the incidence of recurrence in the region of mediastinal lymph nodes seem to be comparable with the results of conventional thoracoscopic subtotal esophagectomy [6]. The data in thoracoscopic esophagectomy 70 cases in our institute from 2017 to 2022 was shown as supplementary files: Supplementary table 1, clinical background; Supplementary table 2, surgical and postoperative outcomes; and Supplementary table 3, postoperative complications. Since the majority of robotic-assisted sub-total esophagectomies have been performed at our institution since December 2018, we reviewed 70 cases of thoracoscopic sub-total esophagectomies from 2017 to 2022. The incidence of left recurrent nerve palsy in thoracoscopic surgery was not different from that in robot-assisted surgery, although a simple comparison cannot be made because of the difference in operators and the timing of surgery. In thoracoscopic surgery, the esophagus was suspended from the dorsal side, which may have increased the operation time. In this study, the omission of suspending the esophagus simplifies the procedure and avoids excessive traction on the left recurrent nerve. Anastomotic leakage could be more observed in thoracic surgery, it may be an effect of the change in anastomosis method from 2018 [10]. The time required for thoracic surgery in this study was not inferior to the time required for general thoracoscopic surgery. This may be due to the lack of preparation for esophageal traction, but it may also be due to the advantages of robotic surgery. Multi-joint motion allows LRLN dissection to be performed parallel to the direction of the nerve fiber. Stable organ mobilization and gentle camera work may also be a factor in shortening time. An advantage of robotic surgery in esophageal cancer surgery is the high potential to reduce nerve palsy, which may lead to the prevention of postoperative pneumonia [11, 12].

The present study was associated with two important limitations. Firstly, this study was conducted at a single institution, and a multicenter study would be desirable to obtain more accurate results. Secondly, not all cases of recurrent nerve palsy were evaluated by otolaryngologists. If such evaluations had been conducted, more cases of recurrent nerve palsy may have been detected.

## Conclusions

The natural creation of surgical space for LRLN dissection in RATE may be a promising maneuver to ensure the retrieval of a sufficient number of lymph nodes and may be a safe procedure for preventing recurrent nerve palsy.

## Supplementary Information

**Additional file 1: Fig. S1.** a) The 1st arm is used to traction the esophagus dorsally and the 4th arm is used to gently push down the trachea to separate the connective tissue, including the left recurrent nerve (lt. RN) and the left recurrent lymph node (LRLN), from the left border of the trachea. The lt. SCA indicates left subclavian artery, the TD indicates thoracic duct. A red broken line indicates the resection line. b) Flip up the LRLN from the left recurrent nerve. The trachea is rotated by the assistant at this time. The lt. RN is separated from the surrounding connective tissue. c) The esophagus is separated from the dorsal tissue. The thoracic duct (TD) is preserved on the vascular sheath side. d) The LRLN is located within the membrane connecting the esophagus to the lateral side of the vascular sheath. The esophagus is pulled out toward the white arrow, and resected the membrane from the vascular side. The lt. RN is away from the membrane being pulled out.

**Additional file 2.** Maneuver of lymph node dissection surrounding the left recurrent nerve (video).

**Additional file 3: Table S1.** Clinical background. **Table S2.** Surgical and postoperative outcomes. **Table S3.** Postoperative complications.

## Data Availability

Raw data were generated at the Department of Surgery and Science, Kyushu University. Derived data supporting the findings of this study are available from the corresponding author on request.
