# Supplementary figures and images for: Left recurrent nerve lymph node dissection in robotic esophagectomy for esophageal cancer without esophageal traction
Source: World J Surg Oncol. 2023 Jul 26;21:223. doi: 10.1186/s12957-023-03117-3 (PMC10369715; doi:10.1186/s12957-023-03117-3)

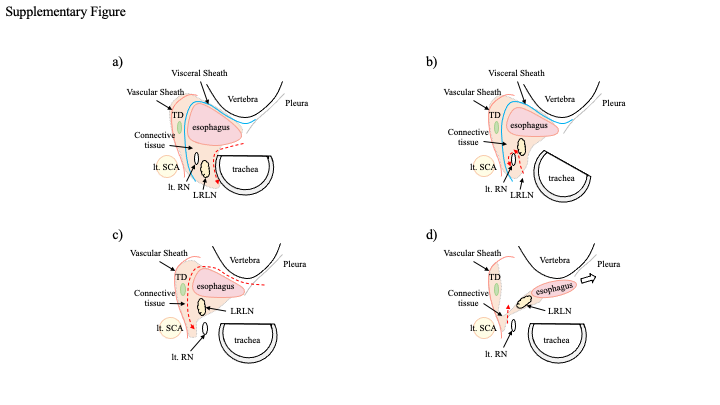

Supplement: Supplementary file 1 — Additional file 1: Fig. S1. a) The 1st arm is used to traction the esophagus dorsally and the 4th arm is used to gently push down the trachea to separate the connective tissue, including the left recurrent nerve (lt. RN) and the left recurrent lymph node (LRLN), from the left border of the trachea. The lt. SCA indicates left subclavian artery, the TD indicates thoracic duct. A red broken line indicates the resection line. b) Flip up the LRLN from the left recurrent nerve. The trachea is rotated by the assistant at this time. The lt. RN is separated from the surrounding connective tissue. c) The esophagus is separated from the dorsal tissue. The thoracic duct (TD) is preserved on the vascular sheath side. d) The LRLN is located within the membrane connecting the esophagus to the lateral side of the vascular sheath. The esophagus is pulled out toward the white arrow, and resected the membrane from the vascular side. The lt. RN is away from the membrane being pulled out. [file 12957_2023_3117_MOESM1_ESM.tiff]
