# Supplementary material for: Left recurrent nerve lymph node dissection in robotic esophagectomy for esophageal cancer without esophageal traction
Source: World J Surg Oncol. 2023 Jul 26;21:223. doi: 10.1186/s12957-023-03117-3 (PMC10369715; doi:10.1186/s12957-023-03117-3)
Supplement: Supplementary file 3 — Additional file 3: Table S1. Clinical background. Table S2. Surgical and postoperative outcomes. Table S3. Postoperative complications. [file 12957_2023_3117_MOESM3_ESM.zip › Supplementary Table 1.docx]

Supplementary Table 1: Clinical Background

|  | Thoracoscopic Esophagectomy  (n=70) |
| --- | --- |
| Age _(range)_ | 67 (44 - 86) |
| Sex _(%)_  Male  Female | 57 (81)  13 (19) |
| Tumor location _(%)_  Upper  Middle  Lower | 17 (24)  32 (46)  21 (30) |
| cT _(%)_  1  2  3  4a | 27 (38)  16 (23)  25 (36)  2 (3) |
| cN _(%)_  0  1  2  3 | 44 (63)  19 (27)  5 (7)  2 (3) |
| cStage _(%)_  I  II  III  IVA | 29 (41)  21 (30)  18 (26)  2 (3) |
| Preoperative treatment  None _(%)_  CT or CRT _(%)_ | 25 (36)  45 (64) |

CT: Chemotherapy, CRT: Chemoradiotherapy
