# Supplementary material for: Left recurrent nerve lymph node dissection in robotic esophagectomy for esophageal cancer without esophageal traction
Source: World J Surg Oncol. 2023 Jul 26;21:223. doi: 10.1186/s12957-023-03117-3 (PMC10369715; doi:10.1186/s12957-023-03117-3)
Supplement: Supplementary file 3 — Additional file 3: Table S1. Clinical background. Table S2. Surgical and postoperative outcomes. Table S3. Postoperative complications. [file 12957_2023_3117_MOESM3_ESM.zip › Supplementary Table 2.docx]

Supplementary Table 2: Surgical and Postoperative Outcomes

|  | Thoracoscopic Esophagectomy  (n=70) |
| --- | --- |
| Operation time _(min)_  Thoracic surgery _(range)_ | 237 (95 – 338) |
| Blood loss _(g) (range)_ | 153 (1 – 450) |
| Number of Lymph node retrieved  Upper mediastinal LNs _(range)_  LRLNs _(range)_ | 9 (1 – 29)  3 (0 – 11) |
| Recurrence in Mediastinal LNs _(%)_ | 9 (13) |

LNs: Lymph Nodes, LRLNs: Left Recurrent Nerve Lymph nodes
