# Supplementary material for: Left recurrent nerve lymph node dissection in robotic esophagectomy for esophageal cancer without esophageal traction
Source: World J Surg Oncol. 2023 Jul 26;21:223. doi: 10.1186/s12957-023-03117-3 (PMC10369715; doi:10.1186/s12957-023-03117-3)
Supplement: Supplementary file 3 — Additional file 3: Table S1. Clinical background. Table S2. Surgical and postoperative outcomes. Table S3. Postoperative complications. [file 12957_2023_3117_MOESM3_ESM.zip › Supplementary Table 3.docx]

Supplementary Table 3: Postoperative Complications

|  | Thoracoscopic Esophagectomy  (n=70) |
| --- | --- |
| All _(%)_ | 27 (38) |
| Pneumonia _(%)_ | 8 (11) |
| Anastomotic leakage _(%)_ | 13 (18) |
| Left recurrent nerve palsy _(%)_ | 5 (7) |
